# Data on the epidemiology of heart failure in Sub-Saharan Africa

**DOI:** 10.1016/j.dib.2018.01.100

**Published:** 2018-02-12

**Authors:** Ulrich Flore Nyaga, Jean Joel Bigna, Valirie N. Agbor, Mickael Essouma, Ntobeko A.B. Ntusi, Jean Jacques Noubiap

**Affiliations:** a Department of Internal Medicine and Specialties, Faculty of Medicine and Biomedical Sciences, University of Yaoundé I, Yaoundé, Cameroon; b Faculty of Medicine, University of Paris Sud XI, Le Kremlin Bicêtre, Paris, France; c Ibal sub-divisional Hospital, Oku, North-west Region, Cameroon; d Department of Medicine, University of Cape Town and Groote Schuur Hospital, Cape Town, South Africa; e Hatter Institute for Cardiovascular Research in Africa, Department of Medicine, University of Cape Town, Cape Town, South Africa; f Cape Universities Body Imaging Center, University of Cape Town, Cape Town, South Africa

**Keywords:** Heart failure, Prevalence, Aetiologies, Treatment, Mortality, Sub-Saharan Africa

## Abstract

In Sub-Saharan Africa (SSA), chronic non-communicable diseases and cardiovascular diseases in particular, are progressively taking over infectious diseases as the leading cause of morbidity and mortality. Heart failure is a major public health problem in the region. We summarize here available data on the prevalence, aetiologies, treatment, rates and predictors of mortality due to heart failure in SSA.

**Specifications Table**

**Table:** 

Subject area	Medicine
More specific subject area	Cardiology
Type of data	Data presented in tables and figures
How data was acquired	Systematic search of literature
Data format	Raw and analyzed data
Experimental factors	Not applicable
Experimental features	Not applicable
Data source location	Not applicable
Data accessibility	All data are included in this article
Related research article	Heart failure in sub-Saharan Africa: a contemporaneous systematic review and meta-analysis. International Journal of Cardiology; In Press

**Value of the data**

– This work provides a deeper understanding of the prevalence, etiologies and prognosis of heart failure in SSA.
– The data allow examination of the different medications used for the treatment of heart failure and therefore could help in changing practices for an optimal management of this pathology.
– The data could be used as a baseline for comparison in future studies.

## 1 Data

In SSA, heart failure is a major public health problem, associated with high morbidity and mortality. Due to the shortage of data to distinctly understand the epidemiology of this pathology in this part of the world, we present here a summary of available data on the prevalence, aetiology, treatment, and prognosis of heart failure in SSA.

## 2 Experimental design, materials, and methods

Through a systematic literature search in MEDLINE and EMBASE (search strategies are presented in Table 1, Table 2), we included all published studies from January 1, 1996 to June 23, 2017 with available data on the prevalence, incidence, aetiologies, diagnosis, treatment and outcomes of heart failure in patients aged 12 years and older, living in SSA. We excluded studies conducted exclusively on African populations living outside Africa, commentaries, editorials, letters to the editor, case reports and case-series of less than 30 participants, studies lacking relevant data to compute the prevalence of the different heart failure aetiologies or treatment, and for duplicate studies, the most comprehensive and/or recent study with the largest sample size was considered, studies with inaccessible full-text, even after request from the corresponding author.

**Table 1:** Main search strategy for PubMed.

Search	Search term	Hits
1	Heart failure [tiab] OR cardiac failure [tiab] OR cardiac insufficiency [tiab] OR heart disease [tiab]	276, 088
2	(((Africa* [tiab] OR Benin [tiab] OR Botswana [tiab] OR "Burkina Faso" [tiab] OR Burundi [tiab] OR Cameroon [tiab] OR "Canary Islands" [tiab] OR "Cape Verde" [tiab] OR "Central African Republic" [tiab] OR Chad [tiab] OR Comoros [tiab] OR Congo [tiab] OR "Democratic Republic of Congo" [tiab] OR Djibouti [tiab] OR "Equatorial Guinea" [tiab] OR Eritrea [tiab] OR Ethiopia [tiab] OR Gabon [tiab] OR Gambia [tiab] OR Ghana [tiab] OR Guinea [tiab] OR "Guinea Bissau" [tiab] OR "Ivory Coast" [tiab] OR "Cote d'Ivoire" [tiab] OR Jamahiriya [tiab] OR Kenya [tiab] OR Lesotho [tiab] OR Liberia [tiab] OR Madagascar [tiab] OR Malawi [tiab] OR Mali [tiab] OR Mauritania [tiab] OR Mauritius [tiab] OR Mayotte [tiab] OR Mozambique [tiab] OR Namibia [tiab] OR Niger [tiab] OR Nigeria [tiab] OR Principe [tiab] OR Reunion [tiab] OR Rwanda [tiab] OR "Sao Tome" [tiab] OR Senegal [tiab] OR Seychelles [tiab] OR "Sierra Leone" [tiab] OR Somalia [tiab] OR "South Africa" [tiab OR "St Helena" [tiab] OR Swaziland [tiab] OR Tanzania [tiab] OR Togo [tiab] OR Uganda [tiab] OR Zaire [tiab] OR Zambia [tiab] OR Zimbabwe [tiab] OR "Central Africa" [tiab] OR "Central African" [tiab] OR "West Africa" [tiab] OR "West African" [tiab] OR "Western Africa" [tiab] OR "Western African" [tiab] OR "East Africa" [tiab] OR "East African" [tiab] OR "Eastern Africa" [tiab] OR "Eastern African" [tiab] OR "South African" [tiab] OR "Southern Africa" [tiab] OR "Southern African" [tiab] OR "sub Saharan Africa" [tiab] OR "sub Saharan African" [tiab] OR "subSaharan Africa" [tiab] OR "subSaharan African" [tiab]) NOT ("guinea pig" [tiab] OR "guinea pigs" [tiab] OR "aspergillus niger [tiab]"))) AND (Heart failure [tiab] OR cardiac failure [tiab] OR cardiac insufficiency [tiab] OR heart disease [tiab])	
3	#1 AND #2	5012
4	#3 AND **Search limits**: From 1 January 1996 to 10 Oct 2017	2125

**Table 2:** Main search strategy for EMBASE.

#1	‘Heart failure’ OR ‘cardiac failure’ OR ‘cardiac insufficiency’ OR ‘heart disease’	658,990
#2	'africa':ab,ti OR 'algeria':ab,ti OR 'angola':ab,ti OR 'benin':ab,ti OR 'botswana':ab,ti OR 'burkina faso':ab,ti OR 'burundi':ab,ti OR 'cameroon':ab,ti OR 'canary islands':ab,ti OR 'cape verde':ab,ti OR 'central african republic':ab,ti OR 'chad':ab,ti OR 'comoros':ab,ti OR 'congo':ab,ti OR 'democratic republic of congo':ab,ti OR 'djibouti':ab,ti OR 'egypt':ab,ti OR 'equatorial guinea':ab,ti OR 'eritrea':ab,ti OR 'ethiopia':ab,ti OR 'gabon':ab,ti OR 'gambia':ab,ti OR 'ghana':ab,ti OR 'guinea':ab,ti OR 'guinea bissau':ab,ti OR 'ivory coast':ab,ti OR 'cote d ivoire':ab,ti OR 'jamahiriya':ab,ti OR 'kenya':ab,ti OR 'lesotho':ab,ti OR 'liberia':ab,ti OR 'libya':ab,ti OR 'madagascar':ab,ti OR 'malawi':ab,ti OR 'mali':ab,ti OR 'mauritania':ab,ti OR 'mauritius':ab,ti OR 'mayotte':ab,ti OR 'morocco':ab,ti OR 'mozambique':ab,ti OR 'namibia':ab,ti OR 'niger':ab,ti OR 'nigeria':ab,ti OR 'principe':ab,ti OR 'reunion':ab,ti OR 'rwanda':ab,ti OR 'sao tome':ab,ti OR 'senegal':ab,ti OR 'seychelles':ab,ti OR 'sierra leone':ab,ti OR 'somalia':ab,ti OR 'south africa':ab,ti OR 'st helena':ab,ti OR 'sudan':ab,ti OR 'swaziland':ab,ti OR 'tanzania':ab,ti OR 'togo':ab,ti OR 'tunisia':ab,ti OR 'uganda':ab,ti OR 'western sahara':ab,ti OR 'zaire':ab,ti OR 'zambia':ab,ti OR 'zimbabwe':ab,ti OR 'central africa':ab,ti OR 'central african':ab,ti OR 'west africa':ab,ti OR 'west african':ab,ti OR 'western africa':ab,ti OR 'western african':ab,ti OR 'east africa':ab,ti OR 'east african':ab,ti OR 'eastern africa':ab,ti OR 'eastern african':ab,ti OR 'north africa':ab,ti OR 'north african':ab,ti OR 'northern africa':ab,ti OR 'northern african':ab,ti OR 'south african':ab,ti OR 'southern africa':ab,ti OR 'southern african':ab,ti OR 'sub saharan africa':ab,ti OR 'sub saharan african':ab,ti OR 'subsaharan africa':ab,ti OR 'subsaharan african':ab,ti	408,647
#3	#1 AND #2	4165
#4	#3 AND **Search limits**: From 1 January 1996 to 10 Oct 2017	1660

**Fig. 1:**
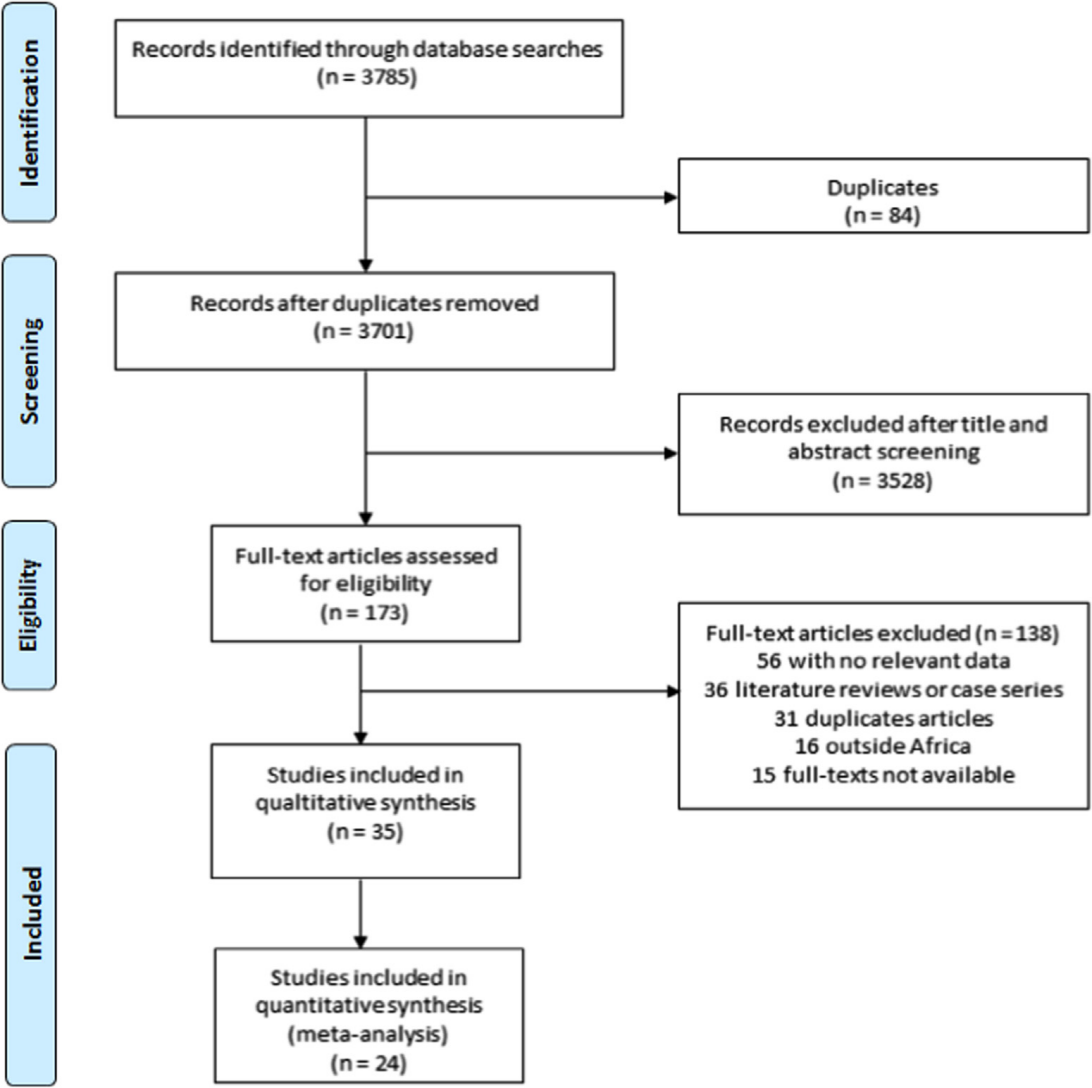
PRISMA flow chart of study selection.

The titles and abstracts of articles retrieved from the bibliographic searches were independently screened by two investigators and full-texts of potentially eligible studies were retrieved and assessed for final inclusion. All discrepancies the selection of studies were resolved through discussion or with the arbitrage of a third investigator. A total of 35 studies were included in this review [1], [2], [3], [4], [5], [6], [7], [8], [9], [10], [11], [12], [13], [14], [15], [16], [17], [18], [19], [20], [21], [22], [23], [24], [25], [26], [27], [28], [29], [30], [31], [32], [33], [34], [35]. A summary of the selection process is presented in the Fig. 1.

Data were then extracted using a predesigned data extraction form. The extracted data include: the last name of first author and the year of study publication, the country in which the study was conducted, Region (Western, Southern, Central, Eastern), area (urban, semi-urban or rural), study design (cross-sectional, cohort, case control), data collection (prospective versus retrospective), random sampling (yes versus no), study population, male proportion, mean or median age (in years), age range (in years), sample size, criteria used for the diagnosis of heart failure, number of cases of the different aetiologies of heart failure and number of cases of the different medications used for the treatment of heart failure.

The quality and risk of bias of all included studies are presented in Table 3, Table 4, Table 5, Table 6, Table 7. It was assessed using the risk of bias assessment tool for developed by Hoy et al. [36]. This tool was adapted for the different topics on heart failure covered in this review (prevalence, aetiology, treatment and prognosis of heart failure).

**Table 3:** Summary table of included studies reporting on heart failure in sub-Saharan Africa (1996–2017).

First name of author, publication year	Country	Region	Area	Study design	Study setting	Data collection	Study population	Random sampling	Male (%)	Mean age (in years)	Age range (in years)	Sample size	Criteria for diagnosis of HF
Oyoo, 1999 [1]	Kenya	Eastern	Urban	Cross-sectional	Hospital-based	Prospective	Patients ≥13 years admitted for congestive heart failure	No	48.4	NR	≥13	91	NR
Thiam, 2003 [2]	Senegal	Western	Urban	Cross-sectional	Hospital- based	Prospective	Patients suffering from heart failure	No	NR	50.0	12–91	170	NR
Kingue, 2005 [3]	Cameroon	Central	Urban	Cross-sectional	Hospital-based	Retrospective and prospective	Patients presenting with clinical and echocardiographic signs of heart failure	No	59.3	57.3	≥16	167	NR
Familoni, 2007 [4]	Nigeria	Western	Semi-urban	Cross-sectional	Hospital-based	Prospective	Patients presenting with acute heart failure	No	61.7	57.6	NR	82	NR
Owusu, 2007 [5]	Ghana	Western	Urban	Cross-sectional	Hospital-based	Prospective	Patients above 12 years admitted with diagnosis of heart failure	No	51.5	51.1	13–90	167	Framingham's criteria
Stewart, 2008 [6]	South Africa	Southern	Urban	Cross-sectional	Hospital-based	Prospective	Novo presentations in patients with heart failure and related cardiomyopathies	No	43	55.0	NR	884	European Society of Cardiology (ESC) guidelines on HF
Ogah, 2008 [7]	Nigeria	Western	Urban	Cross-sectional	Hospital-based	Retrospective	All cases of echocardiography done in the department of medicine between September 2005 and February 2007	No	51.6	54.0	15–90	1441	NR
Onwuchekwa, 2009 [8]	Nigeria	Western	NR	Cross-sectional	Hospital-based	Retrospective	Congestive cardiac failure cases admitted and/or discharged from the medical wards	No	57.2	54.4	18–100	423	Framingham's criteria
Maro, 2009 [9]	Tanzania	Eastern	Urban	Cohort	Hospital-based	Prospective	Patients admitted for congestive heart failure	No	55.0	NR	NR	390	Framingham's criteria
Damasceno, 2012 [10]	The THESUS-HF registry	SSA	–	Cohort	Hospital-based	Prospective	Patients admitted with acute heart failure	No	49.2	52.3	˃12	1006	European Society of Cardiology (ESC) guidelines on HF
Chansa, 2012 [11]	Zambia	Southern	Urban	Cohort	Hospital-based	Prospective	Adult patients (>18 years) admitted for acute heart failure	No	41	50	>18	390	European Society of Cardiology guidelines on HF
Kwan, 2013 [12]	Rwanda	Eastern	Rural	Cross-sectional	Hospital-based	Retrospective	Heart failure patients treated between November 2006 and march 2011	No	30.0	NR	NR	138	NR
Massouré, 2013 [13]	Djibouti	Eastern	NR	Cohort	Hospital-based	Prospective	Djiboutian adults hospitalized for heart failure	No	84.0	55.8	27–75	45	Framingham's criteria
Ojji, 2013 [14]	Nigeria	Western	Urban	Cross-sectional	Hospital-based	Prospective	Subjects of African descent with novo presentations of heart disease	No	49.3	49.0	NR	1515	European Society of Cardiology guidelines on HF
Sliwa, 2013 [15]	The THESUS-HF registry	SSA	–	Cohort	Hospital-based	Prospective	Patients presenting with acute heart failure	No	49.1	52.3	>12	1006	European Society of Cardiology guidelines on HF
Makubi, 2014 [16]	Tanzania	Eastern	Urban	Cohort	Hospital-based	Prospective	Patients ≥ 18 years of age with heart failure defined by the Framingham criteria	No	49.0	55.0	≥18	427	Framingham's criteria
Ogah, 2014 [17]	Nigeria	Western	Urban	Cross-sectional	Hospital-based	Prospective	Patients presenting with acute heart failure	No	54.9	56.4	NR	452	Framingham's criteria and ESC
Pio, 2014 [18]	Togo	Western	Urban	Cross-sectional	Hospital-based	Prospective	Hospitalized patients with heart failure	No	48.2	52.2	18–106	297	European Society of Cardiology guidelines on HF
Pio, 2014 [19]	Togo	Western	Urban	Cross-sectional	Hospital-based	Retrospective	Files of patients hospitalized with heart failure	No	NR	36.5	18–45	376	NR
Osuji, 2014 [20]	Nigeria	Western	NR	Cross-sectional	Hospital-based	Retrospective	All medical admission	No	50.5	60.7	18–110	537	NR
Okello, 2014 [21]	Uganda	Eastern	NR	Cohort	Hospital-based	Retrospective	Patients admitted for acute heart failure	No	30.3	52	NR	274	NR
Dokainish, 2015 [22]	The INTER-CHF registry	SSA	–	Cohort	Hospital-based	Prospective, international, multicenter	Ambulatory and hospitalized adult patients with heart failure	Yes	51.8	53.4	≥18	1294	Boston criteria of HF
Adeoti, 2015 [23]	Nigeria	Western	Urban	Cross-sectional	Hospital-based	Retrospective	All medical admissions	No	55.0	50.9	16–102	3750	NR
Ansa, 2016 [24]	Nigeria	Western	NR	Cross-sectional	Hospital-based	Retrospective medical record review	All cardiovascular admissions to the medical wards	No	NR	NR	≥18	144	NR
Abebe 2016 [25]	Ethiopia	Eastern	Urban	Chart review	Hospital-based	Retrospective	Medical records of patients admitted for heart failure	NR	30.2	53.6	NR	311	NR
Ali, 2016 [26]	Ethiopia	Eastern	Urban	Cohort	Hospital-based	Prospective	Adult patients (>18 years) admitted for heart failure	No	50.7	50.9	NR	152	Framingham's criteria
Kingery, 2017 [27]	Tanzania	Eastern	Urban	Cohort	Hospital-based	Prospective	Medical inpatients admitted for heart failure	No	44.1	52.0	≥18	145	Framingham's criteria
Boombhi, 2017 [28]	Cameroon	Central	Urban	Cross-sectional	Hospital-based	Retrospective	Patients hospitalized for acute heart failure, diagnosed on clinical and/or ultrasound evidence	No	42.7	61,5	16–95	148	NR
Traore, 2017 [29]	Ivory Coast	Western	Urban	Cross-sectional	Hospital-based	Retrospective	Patients hospitalized for heart failure	No	51.0	NR	NR	257	NR
Bonsu, 2017 [30]	Ghana	Western	Urban	Cohort	Hospital-based	Retrospective	Individuals aged ≥ 18 years discharged from first heart failure admission	No	45.6	60.3	≥18	1488	Framingham's criteria
Mwita, 2017 [31]	Botswana	Southern	Urban	Cohort	Hospital-based	Prospective	Patients admitted with acute heart failure	No	53.9	54.2	20–89	193	NR
Pallangyo 2017 [32]	Tanzania	Eastern	Urban	Cohort	Hospital-based	Prospective	Adult patients (>18 years) admitted for heart failure	No	43.5	46.4	>18	463	Framingham's criteria
Sani, 2017 [33]	The THESUS-HF registry	SSA	–	Cohort	Hospital-based	Prospective	Patients presenting with acute heart failure	No	49.2	52.3	>12	954	European Society of Cardiology guidelines on HF
Ogah, 2014 [34]	Nigeria	Western	Urban	Cohort	Hospital-based	Prospective	Patients followed up for heart failure	No	53.1	58.0	NR	239	NR
Carlson, 2017 [35]	Kenya; Uganda	Eastern	NR	Cross sectional	Hospital-based	Prospective	Health facilities with available diagnostic technologies for HF diagnosis	No	NA	NA	NA	340 health facilities (197 in Uganda and 143 in Kenya)	NA

HF=Heart failure; THESUS-HF=sub-Saharan Africa Survey for Heart Failure; INTER-CHF=INTERnational Congestive Heart Failure; NR=Not reported; NA=Not applicable; SSA=Sub-Saharan Africa.

**Table 4:** Summary tables for studies reporting on the prevalence of heart failure sub-Saharan Africa.

First name of author, publication year	Country	Region	Area	Study design	Study setting	Data collection	Random sampling	Population	Male (%)	Mean age	Age range (in years)	Sample size	HF diagnostic tool	Prevalence of HF (%)	Study quality
Osuji, 2014 [20]	Nigeria	Western	NR	Cross-sectional	Hospital-based	Retrospective	No	Patients admitted to the medical ward	50.5	60.7	18–110	537	NR	30.9	Moderate
Kingue, 2005 [3]	Cameroon	Central	Urban	Chart review	Hospital-based	Retrospective	No	Patient >16 years admitted for cardiac pathologies	59.3	57.3	NR	144	Echocardiography	30	Moderate
Ansa, 2016 [24]	Nigeria	Western	Urban	Cross-sectional	Hospital-based	Retrospective	No	All cases of medical admissions	38.9	55	47–65	339	NR	42.5	Low
Pio, 2014 [18]	Togo	Western	Urban	Cross-sectional	Hospital-based	Retrospective	No	Patients admitted to the cardiology unit	NR	52.2	18–106	297	Echocardiagraphy	25.6	High
Pio, 2014 [19]	Togo	Western	Urban	Cross-sectional	Hospital-based	Retrospective	No	Patients admitted to the cardiology unit	NR	36.5	18–45	376	Echocardiagraphy	28.6	Low
Ogah, 2014 [17]	Nigeria	Western	Urban	Cohort	Hospital-based	Prospective	No	All medical admission	54.9	56.4	NR	452	Echocardiagraphy	9.4	High
Adeoti, 2015 [23]	Nigeria	Western	Urban	Cross-sectional	Hospital-based	Retrospective	No	All medical admissions	55.0	50.9	16–102	3750	NR	11.0	Moderate

NR=Not reported.

**Table 5:** Aetiologies of heart failure across sub-Saharan Africa (1996–2017).

First name of author, publication year	Country	Region	Area	Study design	Study setting	Data collection	Study population	Random sampling	Male (%)	Mean age (in years)	Age range (in years)	Sample size	Criteria for diagnosis of HF	Aetiology of heart failure	Diagnostic criteria of IHD	Study quality
Oyoo, 1999 [1]	Kenya	Eastern	Urban	Cross-sectional	Hospital-based	Prospective	Patients ≥13 years admitted for congestive heart failure	No	48.4	NR	≥13	91	NR	Rheumatic heart disease (32%); Cardiomyopathy (25.2%); Hypertensive heart disease (17.6%), pericardial disease (13.2%); Cor pulmonale (7.7%); Ischaemic heart disease (2.2%); Congenital heart disease (2.2%).	ECG and 2D Doppler Echocardiography	Moderate
Thiam, 2003 [2]	Senegal	Western	Urban	Cross-sectional	Hospital- based	Prospective	Patients suffering from heart failure	No	NR	50.0	12–91	170	NR	Hypertension heart disease (34%); Valvular heart diseases (45%); Chronic renal failure (14.5%); Ischaemic heart disease (18.9%); Pulmonary embolism with Right heart failure (3.5%) and aetiology unspecified (6%)	Clinical presentation ECG and Echocardiography	High
Kingue, 2005 [3]	Cameroon	Central	Urban	Cross-sectional	Hospital-based	Retrospective and prospective	Patients presenting with clinical and echocardiographic signs of heart failure	No	59.3	57.3	≥16	167	NR	Hypertensive heart disease (54.5%); Cardiomyopathies (26.3%); Rheumatic heart disease (24.6%), Valvular heart diseases (24.6%), Ischaemic heart disease (2.4%).	12-lead ECG and Echocardiography	Moderate
Familoni, 2007 [4]	Nigeria	Western	Semi-urban	Cross-sectional	Hospital-based	Prospective	Patients presenting with acute heart failure	No	61.7	57.6	NR	82	NR	Hypertensive heart disease (43.4%); Dilated cardiomyopathy (28%); Rheumatic heart disease (9.8%), Endomyocardial fibrosis (2.2%); Cor pulmonale (3.7%); Ischaemic heart disease (8.5%); others (3.5%)	NR	Moderate
Owusu, 2007 [5]	Ghana	Western	Urban	Cross-sectional	Hospital-based	Prospective	Patients above 12 years admitted with diagnosis of heart failure	No	51.5	51.1	13–90	167	Framingham criteria	Hypertensive heart disease (42.5%); Rheumatic heart disease (21.6%); Dilated cardiomyopathy (17.4%); pericardial disease (4.2%); Ischaemic heart disease (3.6%); Cor pulmonale (2.4%) and Congenital heart disease (2.4%)	12-lead ECG and Echocardiography	High
Stewart, 2008 [6]	South Africa	Southern	Urban	Cross-sectional	Hospital-based	Prospective	Novo presentations in patients with heart failure and related cardiomyopathies	No	43	55.0	NR	884	ESC	Dilated cardiomyopathy (35%); Hypertensive heart disease (33%); Right heart failure (27%); Ischaemic heart disease (9%) and Valvular heart disease (8%)	12-lead ECG; echocardiography; stress test; cardiac nuclear imaging and cardiac catheterization	High
Ogah, 2008 [7]	Nigeria	Western	Urban	Cross-sectional	Hospital-based	Retrospective	All cases of echocardiography done in the department of medicine between September 2005 and February 2007	No	51.6	54.0	15–90	1441	NR	Hypertensive heart disease (56.7%); Rheumatic heart disease (3.7%); Dilated cardiomyopathy (3.0%); Pericardial disease (1.8%); cor pulmonale (1.6%); Ischaemic heart disease (0.6%); Congenital heart disease (0.4%); diabetic heart disease (0.4%); thyroid heart disease (0.1%); Sickle cell cardiopathy (0.1%).	NR	High
Onwuchekwa, 2009 [8]	Nigeria	Western	NR	Cross-sectional	Hospital-based	Retrospective	Congestive cardiac failure cases admitted and/or discharged from the medical wards	No	57.2	54.4	18–100	423	Framingham criteria	Hypertensive heart disease (56.3%); Cardiomyopathies (12.2%); Chronic renal failure (7.80%); Severe anemia (4.72%); Rheumatic heart diseases (4.26%). Cor pulmonale (2.13%); Congenital valvular heart disease (0.24%); Ischemic heart disease (0.24%); Missing (11.11%)	12-lead ECG; echocardiography	Moderate
Damasceno, 2012 [10]	The THESUS-HF registry	SSA	–	Cohort	Hospital-based	Prospective	Patients admitted with acute heart failure	No	49.2	52.3	˃12	1006	European Society of Cardiology (ESC) guidelines on HF	Hypertensive heart disease (45.4); Idiopathic dilated cardiomyopathy (18.8%); Rheumatic heart disease (14.3%); Ischaemic heart disease (7.7%); Peripartum cardiomyopathy (7.7%); Pericardial tamponade (6.8%); HIV cardiomyopathy (2.6%); Endomyocardial fibrosis (1.3%).	12-lead ECG; echocardiography; stress test	Moderate
Kwan, 2013 [12]	Rwanda	Eastern	Rural	Cross-sectional	Hospital-based	Retrospective	Heart failure patients treated between November 2006 and march 2011	No	30.0	NR	NR	138	NR	Dilated cardiomyopathy (54%), Rheumatic heart disease (25%), hypertensive heart disease (8%) and ischaemic heart disease (0%)	NR	Moderate
Massouré, 2013 [13]	Djibouti	Eastern	NR	Cohort	Hospital-based	Prospective	Adults hospitalized for heart failure	No	84.0	55.8	27–75	45	Framingham criteria	Coronary artery disease (62%); hypertensive heart disease (18%); rheumatic valvular disease (13%) and primary dilated cardiomyopathy (7%)	12-lead ECG; echocardiography; stress test	Moderate
Ojji, 2013 [14]	Nigeria	Western	Urban	Cross-sectional	Hospital-based	Prospective	Patients with novo presentations of heart disease	No	49.3	49.0	NR	1515	European Society of Cardiology (ESC) guidelines on HF	Hypertensive heart disease (60.6%); Idiopathic dilated cardiomyopathy (12.0%); Valvular rheumatic heart disease (8.6%); peripartum cardiomyopathy (5.3%); Alcoholic cardiomyopathy (4.2%); Thyrotoxic heart disease (2.9%); right heart failure (2.5%); Ischaemic heart disease (0.4%)	ECG; Cardiac enzymes; Echocardiography	High
Makubi, 2014 [16]	Tanzania	Eastern	Urban	Cohort	Hospital-based	Prospective	Patients ≥18 years of age with heart failure defined by the Framingham criteria	No	49.0	55.0	≥18	427	Framingham criteria	Hypertensive heart disease (45%); Cardiomyopathy (28%); Rheumatic heart disease (12%); Ischaemic heart disease (9%); Others^a^ (6%)	12-lead ECG; echocardiography; angiography	High
Ogah, 2014 [17]	Nigeria	Western	Urban	Cross-sectional	Hospital-based	Prospective	Patients presenting with acute heart failure	No	54.9	56.4	NR	452	Framingham criteria and ESC	Hypertensive heart disease (78.5%); Dilated cardiomyopathy (7.5%); Cor pulmonale (4.4%); Pericardial disease (3.3%); Rheumatic heart disease (2.4%); Ischaemic heart disease (0.4%)	12-lead ECG and Echocardiography	High
Pio, 2014 [18]	Togo	Western	Urban	Cross-sectional	Hospital-based	Prospective	Hospitalized patients with heart failure	No	48.2	52.2	18–106	297	European Society of Cardiology (ESC) guidelines on HF	Hypertensive heart disease (43.1%); Ischaemic heart disease (19.2%); Peripartum cardiomyopathy (11.8%); valvulopathies (11.8%); HIV-related cardiopathy (3.4%); Thyrotoxic heart disease (3%); Cor pulmonale (2.7%); congenital cardiopathies (2.7%); Chronic alcoholism (2%) and idiopathic (5.9%).	ECG; Cardiac enzymes; Echocardiography	High
Pio, 2014 [19]	Togo	Western	Urban	Cross-sectional	Hospital-based	Retrospective	Files of patients hospitalized with heart failure	No	NR	36.5	18–45	376	NR	Hypertensive heart disease (42.8%); Valvulopathies (18.1%); Peripartum cardiomyopathy (15.4%); Idiopathic dilated cardiomyopathy (5.8%); Alcoholic cardiomyopathy (3.2%); IHD (2.7%); Congenital cardiopathy (2.7%); Cor pulmonale (2.1%); thyrotoxic heart failure (1.8%); Pericardial tamponade (1.1%) and HIV-associated myocarditis (1.1%)	ECG; Cardiac enzymes; Echocardiography	Low
Dokainish, 2015 [22]	The INTER-CHF registry	SSA	–	Cohort	Hospital-based	Prospective, international, multicenter	Ambulatory and hospitalized adult patients with heart failure	Yes	51.8	53.4	≥18	1294	Boston criteria of HF	Hypertensive heart disease (35%); Ischaemic cardiomyopathy (20%); Idiopathic dilated cardiomyopathy (14.5%); Valvular rheumatic heart disease (7.2%); Endocrine/metabolic heart disease (5.3%); Vavlular non-rheumatic heart disease (2.3%); Alcohol/drug induced cardiopathy (0.7%); HIV cardiomyopathy (0.7%).	12-lead ECG; echocardiography	Moderate
Ansa, 2016 [24]	Nigeria	Western	NR	Cross-sectional	Hospital-based	Retrospective medical record review	All cardiovascular admissions to the medical wards	No	NR	NR	≥18	144	NR	Hypertensive heart disease (48.6%); dilated cardiomyopathy (35.4%); Anaemia (14.6%) and Rheumatic heart disease (1.4%)	NR	Low
Abebe 2016 [25]	Ethiopia	Eastern	Urban	Chart review	Hospital-based	Retrospective	Medical records of patients admitted for heart failure	NR	30.2	53.6	NR	311	NR	Valvular heart disease (40.8%); Hypertensive heart disease (16.1%); Ischaemic heart disease (15.8%); Dilated cardiomyopathy (12.5%), Cor pulmonale (4.5%); Others (10.3%)	NR	Moderate
Kingery, 2017 [27]	Tanzania	Eastern	Urban	Cohort	Hospital-based	Prospective	Medical inpatients admitted for heart failure	No	44.1	52.0	≥18	145	Framingham criteria of HF	Hypertensive heart disease (42.8%); dilated cardiomyopathy (19.3%); Valvular heart disease (16.6%); cor pulmonale (7.6%); ischaemic heart disease (6.2%); Other causes (7.6%)	12-lead ECG; echocardiography	High
Boombhi, 2017 [28]	Cameroon	Central	Urban	Cross-sectional	Hospital-based	Retrospective	Patients hospitalized for acute heart failure, diagnosed on clinical and/or ultrasound evidence	No	42.7	61,5	16–95	148	NR	Hypertensive heart disease (30.16%); Dilated cardiomyopathy (28.57%); Valvular heart disease (11.90%); Chronic cor pulmonale (8.73%); Ischemic heart disease (6.35%); Pericardial diseases (3.96%); Peripartum cardiomyopathy (3.18%)	12-lead ECG; echocardiography	Low
Traore, 2017 [29]	Ivory Coast	Western	Urban	Cross-sectional	Hospital-based	Retrospective	Patients hospitalized for heart failure	No	51.0	NR	NR	257	NR	Hypertensive heart disease (22.9%); Dilated cardiomyopathy (55.57%); Valvular heart disease (6.76%); Ischemic heart disease (11.23%); Other (9.9%)	Echocardiography ± coronarography	Low

Others^a^=Tuberculosis; HIV-related cardiomyopathy; endomyocardial fibrosis; obstructive pulmonary disease; IHD=Ischaemic heart disease; ECG=Electrocardiography; HF=Heart failure; THESUS-HF=sub-Saharan Africa Survey for Heart Failure; INTER-CHF=INTERnational Congestive Heart Failure; ESC=European Society of Cardiology; NR=not reported.

**Table 6:** Summary of studies reporting on pharmacologic treatment of heart failure in sub-Saharan Africa.

First name of author, publication year	Country	Region	Area	Study design	Study setting	Data collection	Random sampling	Male (%)	Mean age (in years)	Age range (in years)	Sample size	Criteria for diagnosis of HF	Treatment of heart failure	Study quality
Kingue, 2005 [10]	Cameroon	Central	Urban	Cross-sectional	Hospital-based	Retrospective and prospective	No	59.3	57.3	≥16	167	NR	Loop diuretics (90%); angiotensin-converting enzyme inhibitor (ACEI) (64.7%); beta-blockers (19.8%); digoxin (30.5%); aldosterone antagonists (25.5%)	Moderate
Stewart, 2008 [7]	South Africa	Southern	Urban	Cross-sectional	Hospital-based	Prospective	No	43.0	55.0	NR	844	ESC	Loop or thiazide diuretic (68%); ACEI (57.7%); beta-blocker (45.6%); digoxin (19%); aldosterone antagonist (42%); calcium channel blocker (18%)	High
Ogah, 2014 [26]	Nigeria	Western	Urban	Cohort	Hospital-based	Prospective	No	54.9	56.4	NR	452	Framingham criteria and ESC	Loop diuretic (88.1%); ACEI (99.1%); beta-blockers (9.1%) digoxin (72.3%); long-acting calcium-channel blockers (26.8%); combined hydralazine and isosorbide dinitrate (14.4%)	High
Damasceno, 2012 [17]	THESUS-HF Registry	SSA	NR	Cohort	Hospital-based	Prospective	No	49.2	52.3	˃12	1006	ESC	Loop diuretic (79%); ACEI/angiotensin receptor blocker (ARB) (82%); beta-blockers (30%); Digoxin (60%); Aldosterone antagonist (75%);	Moderate
Makubi, 2014 [18]	Tanzania	Eastern	Urban	Cohort	Hospital-based	Prospective	No	49.0	55.0	≥18	427	Framingham criteria	Loop diuretics (88%); ACEI/ARB (92%); β-Blockers (42%); Digoxin (39%); Aldosterone antagonist (72%); Calcium channel blockers (19%); Nitrates (64%); Hydralazine (4%)	High
Dokainish, 2016 [19]	INTER-CHF registry	SSA	Both	Cohort	Hospital-based	Prospective, international, multicenter	No	51.8	53.4	≥18	1294	Boston criteria of HF	Diuretic (93.7%); ACEI/ARB (77.1%); β-Blockers (48.3%); Digoxin (31.9%); Aldosterone Inhibitors (59.4%);	Moderate
Boombhi, 2017 [29]	Cameroon	Central	Urban	Cross-sectional	Hospital-based	Retrospective	No	42.7	61.5	16–96	148	NR	Diuretics (93.2%); ACEI/ARB (50%); Beta-blockers (20.6%)	Low
Bonsu, 2017 [30]	Ghana	Western	Urban	Cohort	Hospital-based	Retrospective	No	45.6	60.3	≥18	1488	Framingham criteria of HF	Diuretics (68.4%); ACEI/ARB (62%); β-Blockers (32.5%); Digoxin (16.3%); Aldosterone antagonist (28%); Calcium channel blockers (44.9%); Nitrates (2.1%)	Low
Mwita, 2017 [31]	Botswana	Southern	Urban	Cohort	Hospital-based	Prospective	No	53.9	54.2	20–89	193	NR	ACEI/ARB (67.4%); β-Blockers (72.1%); Loop diuretics (86%); Digoxin (22.1%); Aldosterone antagonist (59.9%)	Moderate

**Table 7:** Summary of studies reporting on the mortality rate and/or predictors of mortality among heart failure patients in sub-Saharan Africa.

First name of author, publication year	Country	Region	Area	Study setting	Data collection	Random sampling	Study Population	Male (%)	Mean age (in years)	Age range (in years)	Sample size	Duration of follow-up	Mortality rate	Predictor(s) of mortality (HR^*^ or OR^**^)	Study quality
Familoni, 2007 [4]	Nigeria	Western	Semi-Urban	Hospital-based	Prospective	No	Adult patients (>18 years) admitted for acute heart failure	67.1	57.6	NR	82	3 years	3-year mortality rate=67.1%	Age (HR=0.997); Systolic blood pressure (HR=1.002); Congestion score (HR=1.007)	Moderate
Maro, 2009 [9]	Tanzania	Eastern	Urban	Hospital-based	Prospective	No	Patients admitted for congestive heart failure	55.0	NR	NR	360	12 months	360-day mortality rate=21.9%	NR	Moderate
															
Chansa, 2012 [11]	Zambia	Southern	Urban	Hospital-based	Prospective	No	Adult patients (>18 years) admitted for acute heart failure	41	50	NR	390	30 days	In-hospital mortality rate=24.1%	Left ventricular ejection fraction <40% (HR=1.93); NYHA class IV (HR=1.92); Serum urea nitrogen >15 mmol/L (HR=2.10); Haemoglobin levels <12 g/dL (HR=1.34); Systolic blood pressure <115 mmHg (HR=1.92)	Moderate
													30-day mortality rate=35%		
															
Sliwa, 2013 [15]	The THESUS-HF registry	SSA	–	Hospital-based	Prospective	No	Patients presenting with acute heart failure	49.1	52.3	NR	1006	Six months	60-day mortality rate=9.5%	Malignancy (HR=5.04); History of cor pulmonale (HR=2.50); Serum urea nitrogen (HR=1.39); Systolic blood pressure (HR=0.91); Rales (HR=2.18); West region (HR=1.83)	High
													180-day mortality rate=15.0%		
Massouré, 2013 [13]	Djibouti	Eastern	Urban	Hospital-based	Prospective	No	Adult patients (> 18 years) admitted for heart failure	84	55.8	27–75	45	14.4 months	Mortality rate=18.0%	NR	Moderate
Okello, 2014 [21]	Uganda	Eastern	NR	Hospital-based	Retrospective	No	Patients admitted for acute heart failure	30.3	52	NR	274	13 months	In-hospital mortality rate=18.3%	Hypotension on admission (adjusted OR=4.6); Reduced left ventricular ejection fraction (adjusted OR=7.6)	Low
Makubi, 2014 [16]	Tanzania	Eastern	Urban	Hospital-based	Prospective	No	Adult patients (>18 years) with heart failure	49.0	55	>18	427	7 months	22.4 per 100 person-years	Creatinine clearance (HR=0.98); Pulmonary hypertension (HR=2.11); Anaemia (HR=2.27); No formal education (HR=2.34); Inpatient (HR=3.23); Atrial fibrillation (HR=3.37).	High
Ali, 2016 [26]	Ethiopia	Eastern	Urban	Hospital-based	Prospective	No	Adult patients (> 18 years) admitted for heart failure	50.7	50.9	>18	152	9 months	In-hospital mortality rate=3.9%	NR	Low
Abebe, 2016 [25]	Ethiopia	Eastern	Urban	Hospital-based	Retrospective	NR	Adult patients admitted for HF	30.2	53.8	>18	311	25 months	Mortality rate=14.1%	Advanced age (HR=1.05), Hyponatremia (HR=0.91), elevated creatinine levels (HR=1.97), and absence of medication (spironolactone [HR=0.34], ACEI [HR=0.26] and statin [HR=0.19])	Moderate
															
Kingery, 2017 [27]	Tanzania	Eastern	Urban	Hospital-based	Prospective	No	Adult patients (>18 years) admitted for heart failure	38.3	50.8	>18	145	12 months	In-hospital mortality rate=25.2%	Low eGFR (HR=2.94); Proteinuria (HR=2.03).	High
													360-day mortality rate=57.9%		
Bonsu, 2017 [30]	Ghana	Western	Urban	Hospital-based	Retrospective	No	Adult patients (> 18 years) admitted for heart failure	45.6	60.3	>18	1488	5 years	5-year mortality rate=31.7%	Age (HR=1.01); NYHA IV (HR=1.96); Ejection fraction (HR=0.99); LDLC-C (HR=1.1); Chronic kidney disease (HR=1.74); Atrial fibrillation (HR=1.26); Anaemia (HR=1.40); Diabetes mellitus (HR=1.50); Statin (HR=0.70); Aldosterone antagonists (HR=0.81)	High
															
Mwita, 2017 [31]	Botswana	Southern	Urban	Hospital-based	Prospective	No	Adult patients (>18 years) admitted for acute heart failure	53.9	54.2	20–89	193	1 year	In-hospital mortality rate=10.9%	Advanced age; Lower haemoglobin level; Lower eGFR; Lower serioum sodium levels; Higher length of hospital stay; Higher serum creatinine levels; Higher serum urea levels; Higher serum NT-proBNP levels	Moderate
													30-day mortality rate=14.7%		
													180-day mortality rate=30.8%		
Pallangyo 2017 [32]	Tanzania	Eastern	Urban	Hospital-based	Prospective	No	Adult patients (>18 years) admitted for heart failure	43.5	46.4	>18	463	180 days	180-day mortality rate=57.8%	Renal dysfunction (HR=1.9); Severe anaemia (HR=1.8); Hyponatraemia (HR=2.2); Rehospitalisation (HR=4.3); Cardiorenal anaemia syndrome (HR=2.1)	High
															
Sani, 2017 [33]	The THESUS-HF registry	SSA	–	Hospital-based	Prospective	No	Patients presenting with acute heart failure	49.2	52.3	>12	954	180 days	NR	Predictors of mortality within 60 days: Heart rate (HR=1.07); left atrial size (HR=1.00)	Low
														Predictors of mortality within 180 days: Heart rate >80bpm (HR=1.25); left ventricular posterior wall thickness in diastole >9 mm (HR=1.32); Presence of aortic stenosis (HR=3.60)	

HR*=Hazard ratio; OR**=Odd's ratio; NYHA=New York Heart Association; bpm=Beats per minute; NR=Not reported; eGFR=Estimated glomerular filtration rate.

Data were analyzed using the ‘*meta’* package of R software. A random-effects meta-analysis model was used to pool prevalence estimates after stabilization of the variance of the study-specific prevalence using the Freeman-Tukey single arc-sine transformation [37]. The Egger's test was used to assess publication bias which was considered significant if the p-value <0.1. Summary statistics from meta-analyses of prevalence studies on the medications used to treat heart failure in sub-Saharan Africa are presented in Table 8.

**Table 8:** Summary statistics from meta-analyses of prevalence studies on the medications used to treat heart failure in sub-Saharan Africa.

**Treatment**	**N studies**	**N participants**	**% (95% confidence interval)**	**I² (95% confidence interval)**	**H (95% confidence interval)**	**P heterogeneity**	**P Egger test**
**ACEI/ARB**	9	5692	75.5 (64.4–85.1)	98.8 (98.4–99.0)	8.9 (7.8–10.2)	<0.0001	0.879
**Aldosterone antagonists**	6	4925	51.5 (32.4–70.3)	99.4 (99.3–99.6)	13.4 (11.8–15.2)	<0.0001	0.807
**Digoxin**	7	5027	31.5 (19.4–45.0)	98.9 (98.6–99.2)	9.6 (8.3–11.2)	<0.0001	0.924
**Loop diuretics**	9	5692	81.6 (72.7–89.1)	98.4 (97.8–98.8)	7.8 (6.7–9.0)	<0.0001	0.806
**β-Blockers**	9	5692	31.4 (22.6–41.0)	98.1 (97.4–98.6)	7.3 (6.3–8.5)	<0.0001	0.549

ACEI=Angiotensin II enzyme inhibitor; ARB=Angiotensin receptor blocker; N=frequency; CI=confidence interval.

These data are attached to a systematic review and meta-analysis published in the International Journal of Cardiology [38].
